# Bacterial Pneumonia Is Associated With Myocardial Fibrosis and New-Onset Left Ventricular Dysfunction

**DOI:** 10.1016/j.jacadv.2022.100128

**Published:** 2022-11-02

**Authors:** Adil Rajwani, Ruad Perera, Girish Dwivedi, Y.C. Gary Lee, Calvin Sidhu, Shital Amin, Jeanie Leong, Graham S. Hillis, Grant W. Waterer

An abundance of epidemiological data associate pneumonia with new-onset chronic heart failure.^1^ While these observations persist for many years after pneumonia, the association is established within 90 days of discharge and with the greatest relative risk in younger individuals in whom the absolute risk of heart failure would otherwise be very low.^2^ Bacterial invasion of the myocardium may be at least 1 mechanism by which heart failure is precipitated. Translocation of pneumococcal bacteria into the myocardium resulting in cardiac microlesions and fibrosis has been reproducibly demonstrated in mice^3^ and primates^4^ with mechanisms matching those in pneumococcal translocation across the blood-brain barrier in meningitis. There are currently no human studies of this observation however.

In this exploratory study, we evaluated myocardial composition and function after pneumonia. Hospitalized patients with community-acquired pneumonia (CAP) were prospectively recruited after obtaining written consent if they had a positive bacterial culture or elevated plasma procalcitonin >2 μg/L or empyema. Positive respiratory viral nucleic acid amplification was an exclusion criterion, as was a history of connective tissue disorders, coronary artery disease, myocarditis, cardiomyopathy, heart failure, or pre-existing requirement for loop diuretics. Normal left ventricular ejection fraction by echocardiography confirmed by 2 experienced observers during the index admission was required, and high-sensitivity troponin I was measured. Patients subsequently underwent gadolinium contrast-enhanced cardiac magnetic resonance imaging (CMR) at 14 to 21 days after discharge, with left ventricular dysfunction defined as an ejection fraction <50%. Edema imaging was by T2-weighted imaging and T2 mapping, and myocardial composition was assessed by T1-weighted late gadolinium enhancement (LGE) and T1 mapping. LGE was assessed by 2 expert blinded readers separately and required visualization in 2 orthogonal planes. Computed tomography chest was performed to detect persisting consolidation. The study was approved by the institutional ethics committee.

Twenty patients were recruited (Table 1). All received dual-coverage intravenous antibiotics. None required invasive ventilation or vasopressor resuscitation. On CMR, subepicardial or midwall LGE consistent with myocardial fibrosis was readily detectable in 6 (30%) individuals, of whom 1 (5% of total cohort) also had new left-ventricular dysfunction. T1 color maps demonstrated multifocal rather than diffuse fibrosis. Among patients with LGE, myocardial edema was also evident in 2 (33%). Patients without detectable LGE had a normal ejection fraction and no myocardial edema. No patient exhibited subendocardial LGE to suggest myocardial infarction. Of the 6 individuals with LGE, only 1 (17%) had troponin elevation during the index admission; however, a pre-recruitment troponin on admission in this patient was also normal.

**Table 1:** Summary of Patient Characteristics and by Presence or Absence of LGE

	All (N = 20)	LGE Negative (n = 14)	LGE Positive (n = 6)
Age	58 ± 16	56 ± 17	62 ± 15
Male	11 (55%)	7 (50%)	4 (67%)
Streptococcal infection	4 (20%)	2 (14%)	2 (33%)
Empyema	7 (35%)	3 (21%)	4 (67%)
Abnormal CT at 2 wk^a^	10 (50%)	4 (29%)	6 (100%)
History of smoking	9 (45%)	6 (43%)	3 (50%)
Admission heart rate (beats/min)	101 ± 25)	91 ± 19	123 ± 24
Systolic blood pressure (mm Hg)	127 ± 27	124 ± 29	137 ± 21
Pneumonia Severity Index	68 ± 20	63 ± 19	82 ± 16
Procalcitonin (μg/L)	3.7 (1.5-4.8)	2.8 (1.0-4.8)	4.7 (4.6-4.8)
hs Troponin I elevation^b^	4 (20%)	3 (21%)	1 (17%)
Plasma BNP (ng/L)	81 (24-185)	101 (5-229)	81 (44-151)
Peak CRP (mg/L)	352 ± 104	313 ± 93	443 ± 67
LVEF baseline TTE (%)	62 ± 4	62 ± 4	63 ± 6
LVEF convalescent CMR (%)	63 ± 7	64 ± 6	62 ± 9
CMR native T1 (ms)	995 ± 36	982 ± 25	1,026 ± 42
CMR ECV (%)	25.7 ± 4.7	23.4 ± 2.6	30.5 ± 4.8
CMR mean T2 (ms)	46.5 (45.2-48.3)	46.5 (45.7-47.8)	46.6 (44.5-51.4)

Values are mean ± SD, n (%), or median (IQR).

BNP = B-type natriuretic peptide; CMR = cardiac magnetic resonance imaging; CRP = C-reactive protein; CT = computed tomography; ECV = extracellular volume; hs = high sensitivity; LGE = late gadolinium enhancement; LVEF = left ventricular ejection fraction; SD = standard deviation; TTE = transthoracic echocardiography.

a Abnormal CT scan at 2 weeks is defined as persisting consolidation or pleural effusion at convalescent imaging.

b Troponin elevation is defined as a value above the 99th percentile of the laboratory upper reference limit (≥26 ng/L in men and ≥16 ng/L in women).

In this cohort study of 20 individuals with a mean age of 58 years who were carefully selected for the absence of pre-existing heart failure or cardiac disease, the key finding therefore is that bacterial pneumonia was associated with myocardial fibrosis and new-onset left ventricular dysfunction on convalescent CMR in 30% and 5% of the cohort, respectively. These novel data support existing epidemiological associations between pneumonia and new-onset heart failure. If replicated in further large studies, this could have important implications for the postdischarge care of CAP and potentially also for a proportion of individuals with established idiopathic dilated cardiomyopathy. While CMR detects fibrosis and not bacteria, these findings are also congruent with animal studies reporting cardiac fibrosis consequent to pneumococcal translocation into the myocardium.^3^^,^^4^ The small cohort size precludes interrogation for predictors of LGE; however, the convalescent persistence of pleuropulmonary abnormalities in all those with LGE warrants assessment in future studies. Troponin was elevated in only 1 participant with LGE; however, we measured troponin during the index admission only, and serial biomarker measurement during convalescence when left ventricular dysfunction is emerging may have been informative. Murine histologic studies have also documented that “*S. pneumoniae* replication within the heart preceded visual signs of tissue damage in cardiac tissue section”,^3^ and further time-course studies are required to better understand the transition from quiescent invasion to overt myocardial injury and fibrosis.

The major limitation of this study is the small cohort size; however, the novel demonstration of a pathophysiological process in the myocardium justifies further large studies. In the absence of a control group, a confounding influence of hospitalization for acute illness cannot be excluded; however, none of participants required invasive ventilation or inotropic support, and the LGE pattern indicated focal fibrosis rather than a diffuse injury or infarction. No participant had a prepneumonia CMR, and therefore, pre-existing fibrosis cannot be excluded; however, the coexisting myocardial edema in a proportion supports an acute process, as does the instance of new-onset left ventricular dysfunction. Furthermore, an incidental detection of fibrosis in 30% of a cohort without any prior history of cardiac disease is unlikely (compared to a 3.9% incidence in a CMR study of 1,840 healthy volunteers,^5^ albeit not hospitalized). Viral respiratory tract infections can precipitate myocarditis and LGE; however, the incidence of myocarditis after viral pneumonia is low, no patient with LGE had clinical features of myocarditis, most had a negative troponin, and we systematically tested for and excluded patients with viral infection. Finally, we recruited hospitalized patients with the highest probability of bacteremia which precludes extrapolation to all CAP where a lower burden of bacteremia is likely.

In summary, in this pilot study, we report the novel association of bacterial pneumonia with myocardial fibrosis and new-onset left ventricular dysfunction during convalescence. This is consistent with epidemiological studies associating pneumonia with new-onset chronic heart failure and with animal studies reporting fibrosis following bacterial translocation into the myocardium. Further confirmation in large studies is warranted.
